# Prevalence of complementary and alternative medicine use in Brazil: results of the National Health Survey, 2019

**DOI:** 10.1186/s12906-022-03687-x

**Published:** 2022-08-02

**Authors:** Patricia de Moraes Mello Boccolini, Karine de Lima Sírio Boclin, Islândia Maria Carvalho de Sousa, Cristiano Siqueira Boccolini

**Affiliations:** 1 Núcleo de Informação, Políticas Públicas e Inclusão Social, UNIFASE/FMP, Petrópolis, RJ Brazil; 2 Universidade Estácio de Sá, Rio de Janeiro, RJ Brazil; 3 Fundação Oswaldo Cruz, Instituto de Pesquisas Aggeu Magalhães, Pernambuco, Brazil; 4 Fundação Oswaldo Cruz, Instituto de Comunicação e Informação Científica e Tecnológica em Saúde, Rio de Janeiro, RJ Brazil

**Keywords:** Complementary and alternative medicine, Prevalence, Population survey, Brazil

## Abstract

**Background:**

In recent decades, it has been possible to observe an increase in Complementary and Alternative Medicine (CAM) usage globally for both disease prevention and health promotion purposes. we aim to estimate the prevalence of CAM use and analyze associated factors in Brazil.

**Methods:**

Observational study with data from the 2019 National Health Survey that evaluated a sample of Brazilian adults. The outcome was CAM use, such as acupuncture, homeopathy, medicinal plants and herbal medicines, meditation, and yoga in the last 12 months. A logistic regression model with a 99% confidence interval was used to assess factors associated with CAM use.

**Results:**

The prevalence of CAM use in 2019 was 5.2% (CI99% = 4.8–5.6%), the most used modalities: medicinal plants and herbal medicines, with a prevalence of 3.0% (CI99% = 2.7–3.33) followed by: acupuncture 1.4% (CI99% = 1.3–1.6) homeopathy 0.9% (CI99% = 0.7–1.0), meditation 0.7% (CI99% = 0.6–0.8) and yoga 0.4% (CI99% = 0.4–0.5). We observed important geographical differences in CAM use in Brazil, with a higher prevalence in the North Region, 3.7% (CI99% = 2.81–4.75), where herbal medicines were more frequent the in the other regions. After estimating an adjusted model, women, older people, and people with a higher level of education and per capita income were the ones who used all types of CAM the most. The practice of yoga stands out among women 3.6% (CI99% = 2.49–5.28) and among individuals with higher per capita income 7.5% (CI99% = 2.97–18.93); meditation among individuals with higher educational level 13.4% (CI99% = 6.41–28.33) and acupuncture for those who declared regular or poor health 1.9% (CI99% = 1.51–2.39).

**Conclusions:**

We recommend that the Ministry of Health expand CAM access to Unified Health System users and promote health professionals’ conscious and guided use for the Brazilian population.

## Background

The expression Traditional, Complementary and Integrative Medicine, coined by the WHO [1], is known locally in Brazil as Integrative and Complementary Practices in Health and was implemented in the Unified Health System (SUS) through a 2006’s National Law, revised in 2018, that defines 29 practices officially recognized in the scope of the health system [2, 3]. The Brazilian private health system is optional to their citizens and regulated by the National Health Supplementary Agency, reimbursing only homeopathy and acupuncture [4]. In order to compare the results with the international literature we will use the term Complementary and Alternative Medicine (CAM) in this study.

CAM in Brazil originates from complex systems such as Acupuncture, Homeopathy, Ayurveda and can be organized according to treatment methods, which in this case are herbal medicine and medicinal plants, manual care (acupuncture, chiropractic, osteopathy, massage), body-mind therapies (tai chi chuan, yoga, lian gong, meditation, bioenergetics) or support group therapy such as the Brazilian methodology known as integrative community therapy [5].

It is essential to state that indigenous and traditional medicines are not enrolled in the Brazilian CAM definition. The main reason for this is that indigenous public health care in Brazil has a specific primary care model for indigenous populations through multidisciplinary health teams and is organized together with indigenous authorities as a subsystem [5].

Worldwide, the prevalence of CAM use in the general population can range from 10 to 75% [6]. The Brazilian National Health Survey (PNS) is undertaken every 5 years and considering data from the 2013 edition we observed that more than seven million adults reported using CAM, representing a prevalence of 4.5% [7]. When comparing the PNS data with those from other countries, we can observe variability in prevalence estimates: in the United States, the prevalence of CAM use was 33%, in Germany, it was 40%, and in Malaysia, it was 56% [7–9]. Thus, this article aims to estimate the prevalence of CAM use in the Brazilian adult population in 2019, and analyze factors associated with CAM use in Brazil.

## Methods

### Study design, objective, and sampling

This study is a cross-sectional survey using a representative sample of the Brazilian adult population residing in permanent households in Brazil, the 2019 National Health Survey (PNS-2019). We obtained the secondary data from the “Instituto Brasileiro de Geografia e Estatística” regarding the “CAM use” chapter from PNS [10].

The PNS-2019 sample consisted of clusters in three stages, with stratification of census tracts (primary sampling units - PSU), where, first, the Primary Analysis Unit were randomly selected, followed by random selection of a fixed number of permanent households. Finally, a resident over 15 years of age or older was randomly selected in that household from a list of eligible residents. Trained interviewers collected data using a structured questionnaire. The survey had three questionnaires, one for the selected resident, one for the household information, and the other to collect data about all residents [11]. We used data only for the 18 years old and above for the present study.

The PNS-2019 visited 108,525 households (with a non-response rate of 8.1%), and 94,114 interviews were carried out (with a non-response rate of 6.4%).[Since this is a sample with a complex design, expansion factors were calculated, including correction factors for losses, followed by weighing and calibration based on population projections.

### Outcome and associated variables

For this study, the outcome was the use of one or more Complementary and Alternative Medicine (CAM) in the last 12 months, which was obtained through the question: “In the last twelve months, (interviewer’s name) used treatments such as acupuncture, homeopathy, medicinal plants and herbal medicine, meditation, yoga, Tai chi chuan, Lian gong or any other integrative and complementary health practice?”. No previous definition of what was meant by integrative and complementary practice was provided to the interviewed person.

For the subjects who answered this question positively, additional questions were asked to detail which CAM was (were) used, namely: Acupuncture, Homeopathy, Medicinal Plants, Meditation, Yoga, Tai chi chuan (or Lian gong, or Qi gong), Community Therapy, or others. Multiple responses were possible. The answers were considered individually and later computed to identify how many CAM modalities among the listed were used by each respondent.

The per capita income variable was calculated and converted to the Brazilian minimum wage. In 2019, the minimum wage in Brazil was R$998.00. This amount was divided by the exchange rate to USD in that same year ($3.946) to make the correspondence of Brazilian minimal wage in USD. We then categorized into up to $126.46 (equivalent to up to ½ minimum wages -MW), over $136.46 and up to $252.91 (over ½ MW and up to 1 MW) - Over $252.91 and up to $502 .8 (More than 1 MW and up to 2 MW) - More than $502.83 (More than 2 MW).

The covariates used in this study were the Brazilian regions in which the respondent lives, sex (male, female), age group (18–39, 40–59, 60 or older), educational level (complete elementary school, complete high school, university degree or more), per capita income (up to ½, more than 1/2 and up to 1, more than one up to 2, and more than two minimum wages -MW), color/race (non-white and white), self-assessment in health (good/very good, fair/bad/very bad), use of private health insurance (no, yes), and access to health care in the last 12 months (no, yes).

We assessed the access to healthcare using the question: “When did (interviewee’s name) last see a doctor?” The answer options were: “Up to one year” (we considered yes, had access to health), “more than one year to 2 years” (we considered as no, did not have access to health), “more than two years to 3 years” (we considered as no, did not have access to health), “more than three years” (we considered as no, did not have access to health), and “never went to the doctor” (we considered as no, did not have access to health). This variable is a proxy for access to the healthcare systemas defined by Boccolini and Souza-Junior (2016) [12].

Initially, we estimated the prevalence of CAM use (one or more CAMs) by categories of sociodemographic characteristics, self-assessment in health, and access to health services. Then, we estimated the association between exposure covariates and the primary outcome (use of one or more CAMs) employing a generalized linear model with a logistic link function and a 99% confidence interval.

In the next step, the prevalence and confidence intervals of 99% of use of the five most frequently used CAMs (Acupuncture, Homeopathy, Medicinal Plants, Meditation, Yoga) were estimated, according to the categories of the covariates or exposure variables, with a confidence interval 99%.

The least used CAMs, such as Integrative and Community Therapy, Tai chi or Lian gong, or Qi gong and Auriculotherapy, were not tabulated.

Then, generalized linear models were estimated, with logistic link function, 99% confidence interval, and 1% alpha, with one model estimated for each of the four most used CAMs and another model for those who used one or more CAMs. All covariates or exposure variables were considered concomitantly in these models, regardless of their statistical significance level.

In all stages, we considered the sample weights and the complex design of the sample. All analyses were conducted using the R version 3.6.0 program.

Finally, to compare the prevalence of CAM use in 2019, we used the previously estimated CAM prevalence data from the 2013 National Health Survey (PNS-2013) [13], incorporating the complex sample design. Since the previous publication about CAM use in Brazil in 2013 used a 95% confidence interval, we decided to use the same confidence interval in the 2019 analysis to compare the prevalence evolution. In the PNS-2013 edition, the participants reported only acupuncture, homeopathy, and medicinal and herbal plants (further details in Boccolini & Boccolini, 2020) [13], limiting the comparison with the PNS - 2019 edition. CAM utilization data from PNS-2013 were compared with those from PNS-2019, considering differences in point prevalence and the absence of interpolation of the 95% confidence intervals.

## Results

The prevalence of CAM use in Brazil in 2019 was 5.2% (CI99% = 4.8–5.6%), being higher in the North and South macro-regions, among female individuals, with higher education, higher income, in older age groups, and of white color/race. The CAM use prevalence was higher among individuals who reported a worse health situation, had a private health plan, and had greater healthcare access (Table 1).

**Table 1:** Prevalence of Complementary and Alternative Medicine (CAM) use, according to sociodemographic characteristics, (Brazil, PNS, 2019) ^e^

Variables	Prevalence (%) ^a^	99%CI ^b^
**Region**		
Southeast	5.0	4.4–5.6
South	6.1	5.3–6.8
Midwest	4.2	3.5–5.0
Northeast	4.9	4.1–5.7
North	6.6	5.7–7.9
**Sex**		
Male	3.8	3.5–4.2
Female	6.4	6.0–6.9
**Age Group**		
18 to 39 years old	4.1	3.7–4.5
40 to 59 years old	5.8	5.3–6.3
60 years old or more	6.5	5.9–7.1
**Educational level**^c^		
Complete Elementary school	4.2	3.7–4.6
Complete High school	3.7	3.3–4.1
University degree or more	9.9	9.0–10.8
**Per capita income**^d^		
Up to ½ MW	3.7	3.1–4.3
Over 1/2 MW and up to 1 MW	3.5	3.1–4.0
Over 1 MW and up to 2 MW	4.4	3.9–5.0
Over 2 MW	10.1	9.1–11.0
**Race**		
Non-white	4.4	4.0–4.8
White	6.2	5.7–6.8
**Self-assessment in health**		
Good/very good	4.7	4.4–5.1
Fair/bad/very bad	6.2	5.6–6.7
**Private Health Insurance**		
No	3.9	3.5–4.3
Yes	8.7	7.9–9.5
**Access to health services**		
No	2.9	2.5–3.3
Yes	5.9	5.5–6.3
**Brazil (total)**	5.2	4.8–5.6

^a^Prevalence considering the complex sample design

^b^99% confidence interval considering the complex sample design

^c^ The variable education categories “illiterate and incomplete elementary school” were aggregated

^d^Per capita income: Up to $126.46 (equivalent to Up to ½ Minimum Wage -MW), over $136.46 and up to $252.91 (over ½ MW and up to 1 MW) - Over $252.91 and up to $502.8 (More than 1 MW and up to 2 MW) - Over $502.83 (More than 2 MW)

^e^Sample of individuals aged 18 years and older who answered the 2019 National Health Survey individual questionnaire

In adjusted analyses, individuals from the South, Northeast, and North regions were more likely to report CAM use when compared to individuals from the Southeast region. No differences in CAM use in the Mid-West region, compared to other regions, were found. Higher chances of CAM use were also found among women, with complete higher education, per capita family income greater than two minimum wages, and aged 40 years or more when compared to the reference categories (male sex, complete elementary school, up to half the minimum wage and between 18 and 39 years of age). Reporting worse health status, having a private health insurance plan, and access to healthcare are also associated with greater CAM use (Table 2).

**Table 2:** Factors associated with the use of Complementary and Alternative Medicine, (Brazil, PNS,2019)^f^

Prevalence of CAM use ^**a**^ (99%CI) ^**b**^ by sociodemographic variables	AOR (%) ^**c**^	99%CI
**Region**		
Southeast	1.00	–
South	1.26	1.05–1.52
Midwest	0.92	0.74–1.15
Northeast	1.32	1.09–1.61
North	1.93	1.59–2.35
**Sex**		
Male	1.00	–
Female	1.57	1.44–1.70
**Age Group**		
18 to 39 years old	1.00	–
40 to 59 years old	1.34	1.20–1.49
60 years old or more	1.42	1.24–1.63
**Educational level**^d^		
Complete Elementary school	1.00	–
Complete High school	1.00	0.88–1.12
University degree or more	1.99	1.72–2.30
**Per capita income**^e^		
Up to ½ MW	1.00	–
Over 1/2 MW and up to 1 MW	0.89	0.75–1.06
Over 1 MW and up to 2 MW	1.03	0.86–1.24
Over 2 MW	1.78	1.44–2.19
**Race**		
Non-white	1.00	–
White	1.10	0.99–1.22
**=Self-assessment in health**		
Good/very good	1.00	–
Fair/bad/very bad	1.59	1.43–1.77
**Private Health Insurance**		
No	1.00	–
Yes	1.47	1.28–1.68
**=Access to health services**		
No	1.00	–
Yes	1.39	1.21–1.60

^a^Prevalence considering the complex sample design

^b^99% confidence interval considering the complex sample design

^c^Adjusted Odds Ratio considering the complex sample design

^d^The variable education categories “illiterate and incomplete elementary school” were aggregated”

^e^Per capita income: Up to $126.46 (equivalent to Up to ½ Minimum Wage -MW), over $136.46 and up to $252.91 (over ½ MW and up to 1 MW) - Over $252.91 and up to $502.8 (More than 1 MW and up to 2 MW) - Over $502.83 (More than 2 MW)

^f^Sample of individuals aged 18 years and older who answered the 2019 National Health Survey individual questionnaire

According to sociodemographic and health characteristics, Table 3 shows the prevalence of the types of CAM most frequently used by the Brazilian population (acupuncture, homeopathy, medicinal plants, meditation, and yoga). The prevalence distribution followed a similar pattern according to the region of residence, being higher in the South and Southeast regions, except medicinal plants, which showed a higher prevalence of use in the North and Northeast regions. We found higher prevalence of CAM use among females, higher income levels, education, and access to health care for all CAMs. Higher prevalence of acupuncture, homeopathy and medicinal plants were also found among individuals aged 60 years and over, and meditation and yoga among younger people (up to 59 years of age).

**Table 3:** Prevalence of of the five most used Complementary and Alternative Medicine (CAM), according to sociodemographic characteristics, (Brazil, PNS, 2019) ^e^

	Acupuncture	Homeopathy	Medicinal Plants	Meditation	Yoga
**Variables**	**Prevalence (%)**^**a**^**(IC99%)**^**b**^	**Prevalence (%) (IC99%)**	**Prevalence (%) (IC99%)**	**Prevalence (%) (IC99%)**	**Prevalence (%) (IC99%)**
**Region**					
Southeast	**2.1** (1.7–2.4)	**1.2** (0.9–1.5)	1.9 (1.5–2.3)	**0.8** (0.6–1.1)	**0.56** (0.4–0.73)
South	1.6 (1.3–1.9)	**1.2** (0.9–1.5)	3.2 (2.6–3.8)	**0.9** (0.7–1.2)	**0.65** (0.5–0.8)
Midwest	1.1 (0.8–1.3)	0.9 (0.6–1.1)	2.6 (1.9–3.2)	0.7 (0.5–0.9)	0.36 (0.2–0.5)
Northeast	0.7 (0.6–0.9)	0.2 (0.2–0.3)	**3.9** (3.2–4.6)	0.3 (0.2–0.4)	0.19 (0.1–0.3)
North	0.4 (0.3–0.6)	0.5 (0.3–0.6)	**5.** (5.0–6.9)	0.2 (0.1–0.3)	0.12 (0.1–0.2)
**Sex**					
Male	0.9 (0.7–1.0)	0.5 (0.4–0.6)	2.4 (2.1–2.7)	0.4 (0.3–0.5)	0.2 (0.1–0.2)
Female	**1.9** (1.7–2.2)	**1.2** (1.0–1.4)	**3.5** (3.1–3.8)	**0.9** (0.7–1.0)	**0.7** (0.5–0.8)
**Age Group**					
18 to 39 years old	1.0 (0.8–1.2)	0.7 (0.5–0.9)	2.3 (1.9–2.5)	**0.7** (0.6–0.9)	**0.5** (0.4–0.6)
40 to 59 years old	1.7 (1.4–2.0)	**1.0** (0.8–1.2)	3.2 (2.8–3.6)	**0.7** (0.5–0.8)	**0.4** (0.3–0.5)
60 years old or more	**1.9** (1.5–2.2)	**1.0** (0.8–1.2)	**4.0** (3.5–4.4)	0.5 (0.3–0.7)	0.3 (0.2–0.8)
**Educational level**^c^					
Complete Elementary school	0.6 (0.5–0.8)	0.3 (0.2–0.4)	3.3 (2.9–3.7)	0.07 (0.0–0.1)	0.04 (0.0–0.1)
Complete High school	1.0 (0.8–1.2)	0.5 (0.4–0.7)	2.1 (1.8–2.4)	0.28 (0.2–0.4)	0.17 (0.1–0.2)
University degree or more	**3.8** (3.3–4.3)	**2.5** (2.1–3.0)	**3.8** (3.2–4.4)	**2.46** (2.0–2.9)	**1.64** (1.3–2.0)
**Per capita income**^d^					
Up to ½ MW	0.2 (0.1–0.3)	0.2 (0.1–0.3)	3.3 (2.7–3.9)	0.10 (0.0–0.2)	0.034 (0.0–0.1)
Over 1/2 MW and up to 1 MW	0.5 (0.4–0.6)	0.3 (0.2–0.5)	2.6 (2.2–3.0)	0.19 (0.1–0.3)	0.081 (0.0–0.1)
Over 1 MW and up to 2 MW	1.2 (1.0–1.5)	0.6 (0.4–0.8)	2.6 (2.2–3.0)	0.41 (0.2–0.7)	0.324 (0.1–0.5)
Over 2 MW	**4.2** (3.6–4.8)	**2.6** (2.0–3.1)	**3.6** (3.0–4.1)	**2.18** (1.8–2.5)	**1.442** (1.2–1.7)
**Race**					
Non-white	0.9 (0.7–1.0)	0.4 (0.3–0.5)	**3.1** (2.8–3.5)	0.4 (0.3–0.4)	0.2 (0.1–0.3)
White	**2.1** (1.8–2.4)	**1.4** (1.1–1.7)	2.8 (2.4–3.2)	**1.0** (0.8–1.3)	**0.7** (0.6–0.9)
**Self-assessment in health**					
Good/very good	1.4 (1.2–1.6)	**1.0** (0.8–1.1)	2.4 (2.1–2.6)	**0.8** (0.6–1.0)	**0.54** (0.4–0.6)
Fair/bad/very bad	**1.5** (1.2–1.8)	0.7 (0.5–0.9)	**4.2** (3.8–4.7)	0.3 (0.2–0.4)	0.20 (0.1–0.3)
**Private Health Insurance**					
No	0.6 (0.5–0.7)	0.4 (0.3–0.5)	2.9 (2.6–3.2)	0.3 (0.2–0.3)	0.19 (0.1–0.2)
Yes	**3.7** (3.2–4.1)	**2.1** (1.7–2.5)	**3.2** (2.7–3.8)	**1.7** (1.4–2.0)	**1.15** (0.9–1.4)
**Access to health services**					
No	0.3 (0.2–0.4)	0.3 (0.2–0.4)	2.3 (1.9–2.7)	0.3 (0.2–0.4)	0.13 (0.1–0.2)
Yes	**1.8** (1.6–2.0)	**1.1** (0.9–1.2)	**3.2** (2.9–3.5)	**0.8** (0.6–0.9)	**0.52** (0.4–0.6)
**Brazil**	**1.4 (1.3–1.6)**	**0.9 (0.7–1.0)**	**3.0 (2.7–3.3)**	**0.7 (0.6–0.8)**	**0.4 (0.4–0.5)**

^a^Prevalence considering the complex sample design

^b^99% confidence interval considering the complex sample design

^c^The variable education categories “illiterate and incomplete elementary school” were aggregated”

^d^Per capita income: Up to $126.46 (equivalent to Up to ½ Minimum Wage -MW), over $136.46 and up to $252.91 (over ½ MW and up to 1 MW) - Over $252.91 and up to $502.8 (More than 1 MW and up to 2 MW) - Over $502.83 (More than 2 MW)

^e^Sample of individuals aged 18 years and older who answered the 2019 National Health Survey individual questionnaire

In **bold**: results statistically significant (*p* < 0.05)

The prevalence of use of integrative community therapy was 0.09% (0.06–0.15), tai-chi (lian gong, qi gong) was 0.06% (0.03–0.13), and auriculotherapy was 0.35% (0.27–0.46), non-tabulated data.

White color/race individuals also showed a higher prevalence of CAM use, except for medicinal plants (Table 4). Acupuncture and medicinal plants were frequent among individuals with a report of poor health, while homeopathy, meditation, and yoga were more frequent among those with a report of better health. In the multivariate analyses, the use of CAM was associated with female gender, higher education, older age (except for meditation and yoga), and higher income (except for medicinal plants). Associations between regions showed different patterns. Using the Southeast region as a reference, residents of the South region had greater chances of using medicinal plants; and those from the Midwest region had lower chances of using acupuncture and a greater chance of using medicinal plants. Residents of the Northeast region had a lower chance of using acupuncture and homeopathy and a greater chance of using medicinal plants. Residents of the North region had a lower chance of using acupuncture, meditation and yoga, and a greater chance of using medicinal plants. There were even greater chances of using homeopathy among white individuals with health insurance and health access; greater chances of acupuncture use among individuals with worse self-reported health, who had health insurance and access to health care; greater chances of using medicinal plants among individuals with worse health status; greater chances of using meditation with private health insurance, and greater chances of using yoga among individuals with access to health care (Table 4).

**Table 4:** Factors associated with the of the five most used Complementary and Alternative Medicine, (Brazil, PNS-2019)^f^

	Acupuncture	Homeopathy	Medicinal Plants	Meditation	Yoga
**Prevalence of CAM use**^**a**^**(99%CI)**^**b**^**by sociodemographic variables**	**AOR**^**c**^**(99%CI)**	**AOR (99%CI)**	**AOR (99%CI)**	**AOR (99%CI)**	**AOR (99%CI)**
**Region**					
Southeast	1.0	1.0	1.0	1.0	1.0
South	0.80 (0.61–1.04)	0.96 (0.68–1.37)	**1.77 (1.33–2.37)**	1.17 (0.82–1.66)	1.18 (0.79–1.75)
Midwest	**0.61 (0.45–0.83)**	0.84 (0.57–1.23)	**1.42 (1.02–1.97)**	0.92 (0.61–1.38)	0.73 (0.46–1.17)
Northeast	**0.66 (0.49–0.89)**	**0.34 (0.23–0.50)**	**2.24 (1.69–2.96)**	0.73 (0.50–1.09)	0.72 (0.43–1.21)
North	**0.42 (0.30–0.58)**	0.73 (0.48–1.10)	**3.65 (2.81–4.75)**	**0.44 (0.26–0.72)**	**0.47 (0.25–0.86)**
**Sex**					
Male	1.0	1.0	1.0	1.0	1.0
Female	**1.97 (1.62–2.39)**	**2.05 (1.69–2.49)**	**1.36 (1.24–1.49)**	**1.95 (1.52–2.51)**	**3.63 (2.49–5.28)**
**Age Group**					
18 to 39 years old	1.0	1.0	1.0	1.0	1.0
40 to 59 years old	**1.53 (1.20–1.96)**	**1.34 (1.04–1.73)**	**1.35 (1.19–1.54)**	0.95 (0.67–1.36)	0.72 (0.48–1.07)
60 years old or more	**1.56 (1.18–2.06)**	**1.35 (0.96–1.88)**	**1.59 (1.33–1.90)**	0.91 (0.56–1.47)	0.79 (0.44–1.43)
**Educational level**^d^					
Complete Elementary school	1.0	1.0	1.0	1.0	1.0
Complete High school	**1.51 (1.06–2.14)**	**1.58 (1.11–2.26)**	0.85 (0.74–0.98)	**2.99 (1.63–5.49)**	**2.60 (1.24–5.45)**
University degree or more	**2.90 (2.01–4.17)**	**4.00 (2.70–5.92)**	**1.49 (1.26–1.77)**	**13.48 (6.41–28.33)**	**11.13 (4.51–27.44)**
**Per capita income**^e^					
Up to ½ MW	1.0	1.0	1.0	1.0	1.0
Over 1/2 MW and up to 1 MW	**1.55 (0.95–2.53)**	1.11 (0.60–2.08)	0.85 (0.69–1.03)	1.24 (0.60–2.60)	1.58 (0.62–3.99)
Over 1 MW and up to 2 MW	**2.69 (1.66–4.17)**	1.27 (0.68–2.36)	0.94 (0.75–1.19)	1.49 (0.66–3.35)	**3.59 (1.38–9.29)**
Over 2 MW	**5.16 (2.93–9.08)**	**2.78 (1.47–5.25)**	1.17 (0.92–1.49)	**3.63 (1.66–7.93)**	**7.50 (2.97–18.93)**
**Race**					
Non-white	1.0	1.0	1.0	1.0	1.0
White	1.15 (0.90–1.45)	**1.51 (1.16–1.96)**	0.96 (0.84–1.10)	1.20 (0.88–1.66)	1.36 (0.90–2.06)
**Self-assessment in health**					
Good/very good	1.0	1.0	1.0	1.0	1.0
Fair/bad/very bad	**1.90 (1.51–2.39)**	1.37 (1.02–1.83)	**1.63 (1.43–1.85)**	0.90 (0.66–1.23)	1.02 (0.62–3.99)
**Private Health Insurance**					
No	1.0	1.0	1.0	1.0	1.0
Yes	**2.24 (1.67–3.01)**	**1.50 (1.06–2.13)**	1.13 (0.93–1.35)	**1.43 (0.99–2.05)**	1.47 (0.85–2.55)
**Access to health services**					
No	1.0	1.0	1.0	1.0	1.0
Yes	**2.59 (1.75–3.85)**	**1.95 (1.30–2.92)**	1.14 (0.97–1.34)	1.40 (0.91–2.16)	**1.82 (1.05–3.14)**

^a^Prevalence considering the complex sample design

^b^99% confidence interval considering the complex sample design

^c^Adjusted Odds Ratio considering the complex sample design

^d^The variable education categories “illiterate and incomplete elementary school” were aggregated”

^e^Per capita income: Up to $126.46 (equivalent to Up to ½ Minimum Wage -MW), over $136.46 and up to $252.91 (over ½ MW and up to 1 MW) - Over $252.91 and up to $502.8 (More than 1 MW and up to 2 MW) - Over $502.83 (More than 2 MW)

^f^Sample of individuals aged 18 years and older who answered the 2019 National Health Survey individual questionnaire

In **bold**: results statistically significant (*p* < 0.05)

Table 5 shows the prevalence of acupuncture, homeopathy, medicinal plants, and the use of at least one CAM for the years 2013 and 2019. The primary trend is an increase in prevalence among the sociodemographic variables evaluated in the period. However, there is stability in the prevalence of CAM in the South and homeopathy in the Northeast region. It is also observed that there is stability in the use of CAMs among individuals with lower educational levels and the use of homeopathy among those of non-white color/race. Homeopathy and all CAM use decreased in the South region. There was also a decrease in the prevalence of medicinal plants use in the South and North region. There was also a slight decrease in the use of medicinal plants among non-white individuals (Table 5).

**Table 5:** Evolution of the prevalence of CAM use (Acupuncture, Homeopathy, Medicinal Plants, and all CAM) according to sociodemographic characteristics for the years 2013 and 2019 (PNS,2013 and PNS,2019)^d^

	Acupuncture		Homeopathy		Medicinal Plants		All CAM	
	Prevalence (%) ^**a**^ (95%CI) ^**b**^		Prevalence (%) (95%CI)		Prevalence (%) (95%CI)		Prevalence (%) (95%CI)	
**Year**	**2013**	**2019**	**2013**	**2019**	**2013**	**2019**	**2013**	**2019**
**Region**								
Southeast	1.5 (1.3–1.9)	2.0 (1.8–2.3)	0.6 (0.5–0.9)	1.2 (1.0–1.5)	1.3 (0.9–1.9)	1.9 (1.6–2.2)	3.8 (3.2–4.5)	5.0 (4.5–5.5)
South	1.0 (0.7–1.4)	1.6 (1.3–1.8)	1.0 (0.7–1.6)	1.2 (1.0–1.4)	3.6 (2.8–4.7)	3.2 (2.7–3.6)	6.1 (5.1–7.3)	6.1 (5.5–6.7)
Midwest	0.7 (0.5–0.9)	1.1 (0.9–1.3)	0.7 (0.5–1.0)	0.9 (0.7–1.1)	2.5 (2.0–3.2)	2.6 (2.1–3.0)	4.1 (3.5–4.9)	4.2 (3.7–4.8)
Northeast	0.3 (0.2–0.4)	0.7 (0.6–0.9)	0.2 (0.1–0.3)	0.2 (0.2–0.3)	3.5 (2.9–4.2)	3.9 (3.3–4.5)	4.1 (3.5–4.8)	4.9 (4.3–5.5)
North	0.1 (0.1–0.3)	0.4 (0.4–0.5)	0.6 (0.4–0.8)	0.5 (0.4–0.6)	6.2 (4.9–7.7)	5.9 (5.2–6.6)	7.0 (5.8–8.5)	6.6 (5.9–7.4)
**Sex**								
Male	0.6 (0.5–0.9)	0.9 (0.8–1.0)	0.5 (0.3–0.6)	0.5 (0.4–0.6)	2.4 (2.1–2.8)	2.4 (2.2–2.6)	3.7 (3.3–4.2)	3.8 (3.5–4.1)
Female	1.3 (1.1–1.5)	1.9 (1.7–2.1)	0.7 (0.6–0.8)	1.2 (1.0–1.3)	2.9 (2.6–3.4)	3.5 (3.2–3.7)	5.2 (4.7–5.7)	6.4 (6.1–6.8)
**Age Group**								
18 to 39 years old	0.5 (0.4–0.7)	1.0 (0.8–1.1)	0.5 (0.4–0.7)	0.7 (0.6–0.8)	2.1 (1.8–2.5)	2.2 (2.0–2.5)	3.4 (3.0–3.9)	4.1 (3.8–4.4)
40 to 59 years old	1.3 (1.0–1.7)	1.7 (1.5–1.9)	0.7 (0.5–0.9)	1.0 (0.8–1.2)	3.1 (2.6–3.6)	3.2 (2.9–3.5)	5.5 (4.9–6.2)	5.8 (5.4–6.2)
60 years old or more	1.4 (1.1–1.7)	1.9 (1.6–2.2)	0.5 (0.3–0.6)	1.0 (0.8–1.1)	3.4 (2.9–4.0)	4.0 (3.6–4.3)	5.4 (4.7–6.1)	6.5 (6.0–7.0)
**Educational level**^c^								
Complete Elementary school	0.3 (0.2–0.5)	0.6 (0.5–0.8)	0.3 (0.2–0.4)	0.3 (0.3–0.4)	3.5 (2.9–4.1)	3.3 (3.0–3.6)	4.2 (3.6–4.9)	4.2 (3.9–4.5)
Complete High school	0.8 (0.6–1.0)	1.0 (0.8–1.1)	0.4 (0.3–0.6)	0.5 (0.4–0.6)	1.9 (1.6–2.2)	2.1 (1.9–2.3)	3.3 (3.0–3.7)	3.7 (3.4–4.0)
University degree or more	3.2 (2.6–3.9)	3.8 (3.4–4.2)	1.8 (1.4–2.3)	2.5 (2.2–2.9)	2.4 (1.9–2.9)	3.8 (3.3–4.2)	8.1 (7.2–9.1)	9.9 (9.2–10.6)
**Race**								
Non-white	0.5 (0.4–0.7)	0.9 (0.8–1.0)	0.4 (0.3–0.5)	0.4 (0.4–0.5)	3.2 (2.8–3.8)	3.1 (2.8–3.4)	4.3 (3.7–4.9)	4.4 (4.1–4.7)
White	1.5 (1.2–1.7)	2.1 (1.9–2.4)	0.8 (0.7–1.0)	1.4 (1.2–1.6)	2.1 (1.8–2.4)	2.8 (2.5–3.1)	4.7 (4.3–5.2)	6.2 (5.8–6.7)
**Brazil (total)**	**1,0 (0,8-1,1)**	**1,5 (1,4-1,7)**	**0,6 (0,5-0,7)**	**1,0 (0,9-1,1)**	**2,7 (2,4-3,0)**	**3,2 (3,0-3,4)**	**4,5 (4,1-4,9)**	**5,2(5,1-5,8)**

^a^Prevalence considering the complex sample design

^b^95% confidence interval considering the complex sample design

^c^The variable education categories “illiterate and incomplete elementary school” were aggregated”

^d^Sample of individuals aged 18 years and older who answered the 2013 and 2019 National Health Survey individual questionnaires

## Discussion

Based on our findings, we extrapolated the results and estimated that 8,500,000 Brazilian adults over 18 years old reported using one or more CAM in 2019, equivalent to 5.2% of the country’s population, an increase of 0.7% in the prevalence of CAM use compared to the PNS-2013 research. Medicinal plants were the most frequently used CAMs in Brazil, with substantial regional differences: while in the Southeast, a more industrialized and urbanized Brazilian region, the use of acupuncture was more frequent, in the Northeast and North regions, that contains most of the Amazon Forest, the use of medicinal plants was more frequently reported by the population. The Brazilian Middle-West region has less population and comprises most of Brazilian crops and cattle, having worse healthcare access than the Southeast and South regions. No differences in CAM use were found in the PNS-2019 study when comparing this region with the others. This heterogeneity was maintained when comparing these findings with those of the PNS-2013.

Brazil has five regions, with significant sociodemographic and health differences between them. The South and Southeast regions are the most densely populated and economically developed, with better health indicators, better access to health services [14], and longer life expectancy [15] than the North, Northeast, and Midwest regions. The North region concentrates more than 80% of the indigenous population [16], likely influencing the higher prevalence of medicinal plants. Paradoxically, the North region has the lowest offer of CAM in primary health care [13]. Indigenous practices and their cosmological perspectives are not incorporated into the Brazilian Policy on Integrative and Complementary Health Practices of the Unified Health System and other practices such as prayers, healers, and midwives [17]. In Brazil, the discussion about the insertion of these traditional practices into policies is still incipient and has not received adequate research support, so we do not know what percentage of the population seeks this type of care [18]. We can assume that if these practices were incorporated into the PNS questionnaire, the prevalence of CAM would be even higher in Brazil.

The present study also observed that female individuals with complete higher education, per capita family income higher than two minimum wages (more than $502.83), or aged 40 years or more were more likely to use one or more CAM. This profile of CAM use is similar to that observed in the United States, Swiss and Australia [18–21]. According to the PNS 2019, the demand for health services, in general, was higher among older women with a high level of education [21]. Some studies have shown that women with higher educational levels and wealthier use more frequently CAM [9, 22, 23], especially at different stages of life, such as during pregnancy, postpartum to climacteric, and menopause [24, 25].

The present study observed that individuals with better access to health care are more likely to use CAM, and in the adjusted analysis, it was possible to observe that these individuals were more likely to use acupuncture, homeopathy, and yoga. These findings suggest possible inequalities in access to health services, as these practices require specialized professionals who might not be available in the Brazilian Unified Health System. In the PNS − 2013, it was observed that acupuncture and homeopathy are among the most used CAMs by those who had a private health plan [13]. In PNS- 2019 the use of CAM among private health plan users was 47% higher than among non-users. However, it was not possible to evaluate if CAM was funded by the healthcare (direct use or reimbursement), by the public health system (SUS), out of pocket, or a combination of those possibilities. In Brazil, private healthcare users can usufruct the public health system facilities and services.

There is still a mismatch between the supply and use of CAM, especially within the Unified Health System. The Ministry of Health of Brazil [26] reports an increase in the supply of CAM in the Unified Health System across the country. However, the use of CAM through the Unified Health System is still relatively low [5, 13, 27]. Studies have shown that the offer in Brazil is dependent on health professionals; that is, there is no specific financing, and the offer depends most on the willingness of the health professional to offer CAM services [27]. Thus, practices that require specific inputs (such as acupuncture) and higher specialization, such as Acupuncture and Homeopathy (a specialty restricted to medical activity in Brazil), continue to be offered less, even after 15 years of National Policy on Integrative and Complementary Practices implementation [27].

Regarding self-rated health, the present survey observed that individuals who reported having regular, poor, or very poor health are more likely to use CAM compared to those who reported having better health status. In the evaluation by type of CAM, it was also possible to observe that those who declared a worse health situation are more likely to use acupuncture or medicinal plants. Similar results associating worse health status and greater use of CAM were observed in other studies [9, 19, 28]. Having a private health insurance plan also increased the chances of using CAM, and similar findings were found in other surveys [17, 29, 30].

Meditation, yoga, tai chi chuan (or lian gong or qi gong), integrative community therapy, and auriculotherapy are CAM were not included in the PNS- 2013 and were included in 2019 after insertion in the National Policy on Integrative and Complementary Practices. The present study observed a low prevalence of tai chi chuan (or lian gong or qi gong), and integrative community therapy. Meditation and yoga also had a low prevalence use among Brazilian adults. A population survey conducted in the United States found a prevalence of 8.9% in yoga among adults, but in the United Kingdom, this prevalence was lower at 1.1% [31, 32]. Regarding meditation, a survey conducted in the United States found a prevalence of 18.6% in adults [33].

The yoga and meditation profile practice stood out in the PNS-2019 among female individuals with private health insurance, access to health services, and reported better self-rated health. Individuals with complete higher education were more than ten times more likely to use these two CAM types than others. We can observe that these are types of CAM used by individuals with higher incomes. Similar results for yoga were observed in studies conducted in the United States, the United Kingdom, and Germany [30, 31].

In Brazil, studies about the prevalence of the practice of yoga and meditation are still scarce; however, these practices are understood as a form of health promotion, disease prevention, and even as a therapeutic action, thus being adopted by the Unified Health System within the scope of primary care in health [34]. Yoga and meditation are the CAM with the fastest-growing supply in the country, with a more than 200% increase in the number of services and the one with the highest number of services offered per capita (2.20/100000 inhabitant) [5].

An extensive systematic review reported a variability by which CAMs are defined and classified in studies [6], making it difficult to compare the use of these practices across populations. The type of sampling and the target population is a factor that can influence research results. Several studies focus on specific populations or subjects, which can generate an overestimation of CAM use. An example is the high prevalence of CAM use in studies with individuals with specific illnesses (especially chronic or terminal illnesses), while we find lower prevalence in population-based studies such as the Brazilian PNS-2013 [25, 35, 36].

Regarding the study limitations, the lack of definition of CAM provided by the interviewer might lead to information bias since the interviewee might not correlate a usual practice or therapy with the CAM term used in the questionnaire [37, 38], which might result in the underestimation of CAM use. Also, the respondents may not remember the use of CAM in the last 12 months, resulting in a small recall bias. In the PNS-2019, a “filter” question was asked in which the respondent had the option of answering “yes” or “no” for the use of eight CAMs previously listed, and if the answer was “yes” to the use of these CAMs, the subsequent questions about each of the eight CAMs were carried out separately. This procedure may have underestimated the prevalence of CAM use in Brazil, which could have been avoided if all eight CAMs selected by the research coordination were read in sequence, allowing the respondent to respond positively or negatively to each type of CAM separately. However, as Unified Health System offers 29 types of CAM, we believe that including all of them in the survey would possibly reduce the information bias, which could reflect a higher prevalence of the use of these therapies.

Another limitation of the study was the non-assessment of the out-of-pocket amount for the use of CAM. Unlike the PNS-2013 analyses, it was impossible to know whether the individual used CAM by Unified Health System, health insurance, or private funding. The PNS − 2019 questionnaire did not cover this information. On the other hand, the strength of the research was its population representation, having reached adults from all socioeconomic strata and allowing the generalization of the results.

## Conclusion

The use of CAM is heterogeneous, with essential differences in the patterns of use between Brazilian regions. Cultural issues can explain these differences and aspects related to the supply and access to health services that provide CAM. Wealthier populations with higher education and access to private health plans use CAM more often that depend on supplies and specialized health professionals, such as acupuncture and homeopathy. The poorest part of the population and with less education use medicinal herbs and herbal medicines more frequently, which have easier access and can often be used without the guidance of health professionals.

## Data Availability

The datasets generated and/or analyzed during the current study are available in the PNS 2019 – Base de Dados Fiocruz, repository, https://www.pns.icict.fiocruz.br/bases-de-dados/.

## Abbreviations

TCIM: Traditional Complementary and Integrative Medicine
CAM: Complementary and Alternative Medicine
PIC: Práticas Integrativas e Complementares em Saúde
WHO: World Health Organization
UHS: Unified Health System
SUS: Sistema Único de Saúde
NPICP: National Policy on Integrative and Complementary Practices in Health – PNPIC Política Nacional de Práticas Integrativas e Complementares em Saúde
PNS: National Health Survey (Pesquisa Nacional de Saúde)
PAU: Primary Analysis Units
IBGE: Brazilian Institute of Geography and Statistics (Instituto Brasileiro de Geografia e Estatística)
OR: Odd Ratio
AOR: Ajusted Odd Ratio
CI: Confidence Interval

## References

[1] World Health Organization (WHO) (2000). General guidelines for methodologies on research and evaluation of traditional medicine.

[2] BRAZIL. Ministry of Health (2006). National Policy for Complementary and Integrative Practices. Ordinance n. 971.

[3] Brazil. Ministry of Health (2018). Health Care Secretariat. Ordinance n. 702, of March 21, 2018. It alters the consolidation ordinance n. 2/GM/MS, of September 28, 2017, to include new practices into the National Policy for Complementary and Integrative Practices.

[4] Brazil. Ministry of Health. Agência Nacional de Saúde Suplementar. Resolução Normativa N° 470, de 09 de julho de 2021. Available at: https://www.ans.gov.br/component/legislacao/?view=legislacao&task=textoLei&format=raw&id=NDA2Mw== Acessed 28 May 2022.

[5] Sousa IMC, Bezerra AFB, Guimarães MB, Benevides IA, Tesser CD. Traditional, complementary and integrative medicine in the Brazilian public health service: opportunities and limitations. Public health and health services research in traditional, complementary and integrative. Health Care. 2019:197–216. 10.1142/9781786346797_0012.

[6] Harris PE, Cooper KL, Relton C, Thomas KJ (2012). Prevalence of complementary and alternative medicine (CAM) use by the general population: a systematic review and update. Int J Clin Pract.

[7] Siti ZM, Tahir A, Farah AI, Fazlin SMA, Sondi S, Azman AH (2009). Use of traditional and complementary medicine in Malaysia: a baseline study. Complement Ther Med.

[8] Clarke TC, Barnes PM, Black LI, Stussman BJ, Nahin RL. Use of yoga, meditation, and chiropractors among U.S. adults aged 18 and over. NCHS Data Brief. 2018;(325):1–8. https://pubmed.ncbi.nlm.nih.gov/30475686/. 30475686

[9] Kemppainen LM, Kemppainen TT, Reippainen JA, Salmenniemi ST, Vuolanto PH (2018). Use of complementary and alternative medicine in Europe: health-related and sociodemographic determinants. Scand J Public Health.

[10] Sistema Integrado de Pesquisas Domiciliares (SIPD). Ministério do Planejamento, Orçamento e Gestão / Instituto Brasileiro de Geografia e Estatística – IBGE. Pesquisa Nacional de Saúde (2019). Available at: https://www.ibge.gov.br/estatisticas/sociais/saude/9160-pesquisa-nacional-de-saude.html?=&t=microdados. Acessed 28 Oct 2021.

[11] Stopa SR, et al. Pesquisa Nacional de Saúde 2019: histórico, métodos e perspectivas. Epidemiologia e Serviços de Saúde [online]. 2020;29(5):e2020315. 10.1590/S1679-49742020000500004 33027428

[12] Boccolini CS, Souza Junior PR (2016). Inequities in healthcare utilization: results of the Brazilian National Health Survey, 2013. Int J Equity Health.

[13] Boccolini PMM, Boccolini CS (2020). Prevalence of complementary and alternative medicine (CAM) use in Brazil. BMC Complement Med Ther..

[14] Viacava F, Porto SM, Carvalho CC, Bellido JG. Desigualdades regionais e sociais em saúde segundo inquéritos domiciliares (Brasil, 1998–2013). Ciência Saúde Coletiva. 2019;24(7). 10.1590/1413-81232018247.15812017. 10.1590/1413-81232018247.15812017 31340291

[15] Camargos MCS, Gonzaga MR, Costa JV, Bomfim WC (2019). Estimativas de expectativa de vida livre de incapacidade funcional para Brasil e Grandes Regiões, 1998 e 2013. Ciência Saúde Coletiva.

[16] Instituto Brasileiro de Geografia e Estatística (IBGE) (2012). Os indígenas no Censo Demográfico 2010, primeiras considerações com base no quesito cor ou raça.

[17] Guimarães MB, Nunes JA, Velloso M, Bezerra A, Sousa MII (2020). As práticas integrativas e complementares no campo da saúde: para uma descolonização dos saberes e práticas. Saúde e Sociedade.

[18] Conboy L, Patel S, Kaptchuk TJ, Gottlieb B, Eisenberg D, Acevedo-Garcia D (2005). Sociodemographic determinants of the utilization of specific types of complementary and alternative medicine: an analysis based on a nationally representative survey sample. J Altern Complement Med.

[19] Klein SD, Torchetti L, Frei-Erb M, Wolf U (2015). Usage of complementary medicine in Switzerland: results of the Swiss health survey 2012 and development since 2007. Plos One.

[20] Reid R, Steel A, Wardle J, Trubody A, Adams J (2016). Complementary medicine use by the Australian population: a critical mixed studies systematic review of utilisation, perceptions and factors associated with use. BMC Complement Altern Med.

[21] Malta DC, Bernal RTI, Gomes CS, Cardoso LCM, Lima MG, Barros MBA (2021). Desigualdades na utilização de serviços de saúde por adultos e idosos com e sem doenças crônicas no Brasil. Pesquisa Nacional de Saúde 2019 [preprint] Rev Bras Epidemiol.

[22] Kristoffersen AE, Stub T, Salamonsen A, Musial F, Hamberg K (2014). Gender differences in prevalence and associations for use of CAM in a large population study. BMC Complement Altern Med.

[23] Clarke TC, Black LI, Stussman BJ, Barnes PM, Nahin RL (2015). Trends in the use of complementary health approaches among adults: United States, 2002-2012. Natl Health Stat Report.

[24] Neel K, Goldman R, Nothnagle M (2019). Integrating doulas into hospital births: provider perceptions of doulas and doula care [22C]. Obstet Gynecol.

[25] Johnson A, Roberts L, Elkins G (2019). Complementary and alternative medicine for menopause. J Evid Based Integr Med.

[26] Brasil, Ministério da Saúde. Relatório de Monitoramento Nacional das Práticas Integrativas e Complementares em Saúde nos Sistemas de Informação em Saúde. 2020 Disponível em: http://observapics.fiocruz.br/oferta-de-pics-cresce-na-atencao-primaria-e-especializada/ Acessado 20 Sept 21.

[27] Barbosa FES, Guimarães MBL, Santos CR, Benjamin AF, Dalcanale BC. Oferta de Práticas Integrativas e Complementares em Saúde na Estratégia Saúde da Família no Brasil. Cadernos de Saúde Pública. 2020;36(1). 10.1590/0102-311X00208818. 10.1590/0102-311X00208818 31939549

[28] Fjær EL, Landet ER, McNamara CL, Eikemo TA (2020). The use of complementary and alternative medicine (CAM) in Europe. BMC Complement Med Ther.

[29] Nahin RL, Barnes PM, Stussman BJ (2016). Insurance coverage for complementary health approaches among adult users: United States, 2002 and 2012. NCHS Data Brief, no 235.

[30] Barnes PM, Powell-Griner E, McFann K, Nahin RL (2004). Complementary and alternative medicine use among adults: United States, 2002. Advance data from vital and health statistics; no 343.

[31] Cramer H (2019). Meditation in Deutschland: Eine national repräsentative Umfrage. Complement Med Res.

[32] Ding D, Stamatakis E (2014). Yoga practice in England 1997-2008: prevalence, temporal trends, and correlates of participation. BMC Res Notes.

[33] Macinko J, Upchurch DM (2019). Factors associated with the use of meditation, U.S. adults 2017. J Altern Complement Med.

[34] Barros NF, Siegel P, Moura SM, Cavalari TA, Silva LG, Furlanetti MR, Gonçalves AV (2014). Yoga e promoção da saúde. Cien Saude Colet.

[35] Kaboli PJ, Doebbling BN, Saag KG, Rosenthal GE. Use of complementary and alternative medicine by older adults with arthritis: a population-based study. Arthritis Rheum. 2001;45:398–403. 10.1002/1529-0131(200108)45:4<398::AID-ART354>3.0.CO;2-I. 10.1002/1529-0131(200108)45:4<398::AID-ART354>3.0.CO;2-I 11501729

[36] Quandt SA, Chen H, Grzywacz JG, Grzywacz JG, Bell RA, Lang W, Arcury TA (2005). Use of complementary and alternative medicine by persons with arthritis: results of the National Health Interview Survey. Arthritis Rheum.

[37] Quandt SA, Verhoef MJ, Arcury TA, Lewith GT, Steinsbekk A, Kristoffersen AE (2009). Development of an international questionnaire to measure use of complementary and alternative medicine (ICAM-Q). J Altern Complement Med.

[38] Arcury TA, Bell RA, Snively BM, Smith SL, Skelly AH, Wetmore LK, Quandt SA (2006). J complementary and alternative medicine use as health self-management: rural older adults with diabetes. Gerontol B Psychol Sci Soc Sci.

